# Sex dependent pleiotropic effects of *Apolipoprotein (ApoE) 4* on executive function in mice and humans

**DOI:** 10.1002/alz.092572

**Published:** 2025-01-03

**Authors:** Madison Longmuir, Aya Arrar, Kate M. Onuska, R. Nathan Spreng, Daniel Palmer, Timothy J Bussey, Lisa M Saksida, Vania F Prado, Marco A.M. Prado, Taylor W. Schmitz, PREVENT‐AD Research Group

**Affiliations:** ^1^ Robarts Research Institute, London, ON Canada; ^2^ Schulich Medicine and Dentistry, London, ON Canada; ^3^ University of Western Ontario, London, ON Canada; ^4^ Montreal Neurological Institute, Montreal, QC Canada; ^5^ McConnell Brain Imaging Centre, Montreal, QC Canada; ^6^ Centre for Studies on Prevention of Alzheimer’s disease (StoP‐AD Centre), Montreal, QC Canada; ^7^ Douglas Mental Health University Institute, Montreal, QC Canada; ^8^ McGill University, Montreal, QC Canada; ^9^ Rodent Cognition Research and Innovation Core, London, ON Canada; ^10^ Western University, London, ON Canada; ^11^ Schulich School of Medicine and Dentistry, London, ON Canada; ^12^ Lawson Health Research Institute, London, ON Canada

## Abstract

**Background:**

*ApoE4* is the strongest genetic risk factor for late onset Alzheimer’s Disease (AD). However, *ApoE4* has also been suggested to exhibit antagonistic pleiotropy, a phenomenon by which some allelic variations of a gene promote fitness during certain periods of life but may be detrimental in others. Previous work suggests that *ApoE4* carriers exhibit superior performance on executive function tasks in early and middle age, while later in life (>70 years) *ApoE4* carriers experience greater cognitive decline across multiple domains. We examined this *ApoE4* antagonistic pleiotropy hypothesis using a cross‐species translational approach, focusing on cognitively unimpaired human adult *ApoeE4* carriers and non‐carriers who were within a relatively younger age range (<70 years). We complement this using knock‐in mouse models that express humanized wild‐type *App*, *MAPT*, and *ApoE3* or *ApoE4* genes. To quantify executive function in both human and mouse, we used cross‐species touchscreen based Continuous Performance Task (CPT) to assess selective attention.

**Method:**

Human participants were recruited from the PREVENT‐AD program at the Douglas Research Centre. Human touchscreen CPT testing was conducted using an MS SurfacePro. Mice were tested with the same task as humans using an operant chamber platform.

**Result:**

Our initial behavioural results suggest that *ApoE4* facilitates attentional processes in both older adults and mouse *ApoE4* carriers, as indexed by discrimination performance on the CPT. Further strengthening this translational pattern, we found that the facilitative effect of *ApoE4* on CPT was more pronounced in female *ApoE4* carriers of both species. In mice, we were able to examine this *ApoE*‐dependent effect on attention longitudinally. Consistent with antagonistic pleiotropy hypothesis, *ApoE4* female mice present a higher CPT performance early in life (6‐9 months of age) when compared to *ApoE*3 mice, but this difference was reduced by 12 months of age.

**Conclusion:**

Taken together, these results support the *ApoE4* antagonistic pleiotropy hypothesis, whereby *ApoE4* carriership confers greater attentional performance early in life in both female humans and mice. These new mouse models, in combination with fully translatable touchscreen tests of cognition, can be used to explore potential mechanisms by which *ApoE4* can affect different aspects of brain function across the life span

